# Assessing Auditory Brainstem Response (ABR) Quality: A Retrospective Review of One Center's Findings

**DOI:** 10.1002/oto2.70056

**Published:** 2024-12-22

**Authors:** Hannaan S. Choudhry, Roman Povolotskiy, Shahin Damji, Yu‐Lan M. Ying, Nicole Raia

**Affiliations:** ^1^ Department of Otolaryngology–Head and Neck Surgery Rutgers New Jersey Medical School Newark New Jersey USA; ^2^ Department of Otolaryngology–Head and Neck Surgery Boston University Chobanian and Avedisian School of Medicine Boston Massachusetts USA; ^3^ University Hospital Department of Audiology Newark New Jersey USA

**Keywords:** ABR guidelines, auditory brainstem response, Otolaryngology, pediatric hearing loss, sensorineural hearing loss

## Abstract

**Objectives:**

Auditory brainstem response (ABR) is the gold standard to assess hearing loss in pediatric patients. Multiple widely accepted ABR protocols with varying parameters are accepted, difference in standards may lead to misdiagnosis or delay in diagnosis and treatment. This study investigates the quality of ABR testing in pediatric patients in addition to changes in diagnoses and management.

**Study Design:**

Retrospective chart review.

**Setting:**

University Hospital, Rutgers New Jersey Medical School.

**Methods:**

Retrospective chart review was conducted for all pediatric patients from 2012 to 2019 who had undergone prior outside ABR testing before presenting to our institution for hearing loss evaluation. The ABR tests were analyzed for completeness following the American Academy of Audiology (AAA), American Speech Language Hearing Association (ASHA), and The Joint Committee on Infant Hearing (JCIH) guidelines. Descriptive statistics on changes in patient diagnoses and interventions after repeat ABR were performed.

**Results:**

80 patients met inclusion criteria. The most common reasons for an incomplete ABR were inadequate components of testing including tone burst bone conduction (85.0%), polarity (82.5%), and tone burst air conduction (48.7%). 77 of the patients who presented required a repeat ABR. 37 repeated ABRs resulted in a change of diagnosis, the most common being from unspecified hearing loss to sensorineural hearing loss (10%). 23 cases had a change in ultimate management.

**Conclusion:**

Incomplete ABR testing may result in misdiagnosis, delay in diagnosis and treatment. Identifying common reasons for incomplete ABR testing may aid Otolaryngologists develop a screening workflow to recognize patients requiring repeat testing.

**Level of Evidence:**

4.

Hearing loss is the most common neonatal sensory disorder in the United States, with incidence of approximately 1.4/1000 screened newborns.^1^ Late detection of hearing loss in children leads to lifelong negative impacts on learning, literacy, and speech‐language development.^1^, ^2^ Such delays in developing these skills can impair educational and psychosocial progress in children. Previous literature has shown that early identification and intervention for children with congenital hearing loss can lead to an increase of 40 percentile points on language expressive measures.^1^ Therefore, newborn hearing screening protocols and interventional programs have been established in many countries; in the United States, the National Institutes of Health has recommended mandatory infant hearing screening.^1^, ^2^

Auditory brainstem response (ABR) is the gold standard to assess hearing loss in pediatric patients 2 years of age and younger, and is the only diagnostic mechanism to diagnose hearing loss in pediatric patients under 6 months.^3^, ^4^ An ABR is a response in the lower brainstem that represents a time‐locked change in the ongoing electroencephalogram (EEG) in response to auditory stimuli.^5^ Specifically, the ABR response is believed to be composed of 5‐time locked waveforms that appear representing the ascending auditory pathway: (I) cochlear (VIII) nerve, (II) cochlear nucleus, (III) superior olivary complex, (IV) lateral lemniscus, and (V) inferior colliculus.^5^ Diagnostic ABRs are used to assess neural synchrony and estimate auditory thresholds.^5^ Studies that assess synchrony change the polarity (positive or negative) of the stimuli to compare responses.^6^ Frequency‐specific stimuli, called tone bursts, are very narrow stimuli centered around a target frequency that are used for threshold estimation to determine the degree of hearing loss via air conduction and the type of hearing loss via bone conduction.^6^

Three professional societies each published well‐known and widely accepted ABR guidelines at the time these ABR exams were performed: American Academy of Audiology (AAA), American Speech‐Language‐Hearing Association (ASHA), and Joint Committee on Infant Hearing (JCIH). Each of the guidelines mentioned provide testing parameters for ABR testing listed in Table 1.^7^, ^8^, ^9^ Depending on the specific guideline followed, the testing parameters vary. Hence, there is no unified consensus on how to perform an ABR among different audiology groups. This lack of consensus could potentially lead to over/under diagnosing hearing loss, underestimating the degree of hearing loss (leading to under amplification), inaccurate type of hearing loss (possibly requiring unnecessary imaging), or misidentification of Auditory Neuropathy Spectrum Disorder (ANSD) when these patients are seen by an Otolaryngologist who is relying on the ABR report generated by the Audiologist.^10^, ^11^, ^12^, ^13^, ^14^ This is especially relevant when ABR testing is done independently by an audiology group based loosely on these society guidelines.

**Table 1:** ABR Guidelines/Protocol Joint Committee on Infant Hearing (JCIH), the American Speech‐Language‐Hearing Association (ASHA), and the American Academy of Audiology (AAA)

ABR guidelines/protocol	Tone burst frequencies	Click air	Polarity
JCIH	At least 2 bone and air	Included	At least a single polarity
AAA	At least 2 bone and air	N/A	N/A
ASHA	At least 2 bone and air	N/A	N/A

Abbreviation: N/A, not applicable to specified guideline/protocol.

Currently, there are no studies demonstrating the quality and standards of ABR testing in pediatric patients. This study investigated ABR testing parameters in New Jersey from outside institutions and described whether they meet the testing protocol standards proposed by the JCIH, ASHA, and AAA for infants and children. Changes in diagnosis from the first to repeat ABR as well as any associated changes in treatment were assessed. We hypothesized that most outside ABRs do not meet the standards set by these professional societies, and this inconsistency would lead to changes in diagnosis and treatment after repeat ABR. Finally, a screening protocol was proposed for Otolaryngologists to use when reviewing ABR results to determine if the testing is considered complete and accurate for interpretation.

## Methods

Approval for the current study was obtained from the Institutional Review Board of Rutgers New Jersey Medical School and University Hospital (UH). A retrospective chart review was conducted with all patient data de‐identified and assigned codes for analysis. The charts of all pediatric patients (younger than 18 years old) with hearing loss that presented to Rutgers/UH Department of Audiology between 2012 and 2019 were reviewed by the pediatric Audiologist. A total of 1021 patient records were reviewed. Patients that did not undergo ABR testing prior to referral were excluded from this study. Patients who underwent an ABR prior to 2008 were excluded since exams were conducted prior to the 2007 JCIH Position Statement. Patients that only underwent ABR testing at the Rutgers/UH Department of Audiology facility were also excluded. Patients who were identified as having undergone ABR testing by an outside licensed Audiologist in New Jersey prior to referral to Rutgers/UH Department of Audiology during this time were included for analysis, with the following demographic variables recorded: age, referral source, prematurity, NICU stay >7 days, status of newborn hearing screening, and degree of hearing loss. ABR testing parameters that were reviewed included number of tone burst frequencies performed, presence of click testing, number and type(s) of polarity used, and the presence of raw waveforms with the report. ABR parameters were determined both by those listed in the report and by reviewing the raw waveforms for information on polarity if available. These testing parameters were then compared to the 2007 JCIH, 2012 ASHA, and 2011 AAA testing protocols (Table 1). For this study, AAA and ASHA were grouped together as one entity (AAA/ASHA) because their testing parameters were identical.

This study defines a complete ABR using the testing parameters defined by each society. For the JCIH testing protocol, an ABR was considered complete if the following was performed: (1) high‐intensity click via air conduction performed with rarefaction and condensing polarities, (2) at least 2 frequency tone bursts via air and bone conduction. For the AAA/ASHA testing protocol, an ABR was considered complete if: (1) at least 2 frequency tone bursts via air and bone conduction were performed. Descriptive statistics were carried out to determine if the outside ABR studies were complete based on ABR protocols. Analysis was also performed to determine which components of the ABR were missing. Descriptive statistics were included to identify any changes in diagnosis and/or treatment from first to second ABR. All analyses and statistics were conducted using Microsoft Excel.

## Results

Eighty patients were included in this study who underwent ABR testing by an outside licensed Audiologist in New Jersey prior to referral to Rutgers/UH Department of Audiology. 62.5% of the pediatric patients in this study were between the ages of 0 to 12 months when they received their first ABR (Table 2). 55% of the patients were referred to Rutgers/UH Department of Audiology after their first ABR performed elsewhere by their pediatricians (Table 3). 87.5% of patients were full‐term and 82.5% of patients did not have a prolonged NICU stay of greater than 7 days. 72.5% of patients did not pass their newborn hearing screening. After completing their first outside ABR testing, 49% of patients had a diagnosis of severe to profound hearing loss, 2.5% of patients had normal hearing, and 17.5% of patients did not have a specified degree of hearing loss.

**Table 2:** Age Ranges of First and Second ABR

	Age at first ABR		Age at second ABR	
Age	N	%	N	%
<1 month	3	3.8	0	0.0
1‐3 months	22	27.5	0	0.0
3‐6 months	11	13.8	11	14.3
6‐12 months	14	17.5	17	22.1
12‐24 months	9	11.3	19	24.7
2‐3 years	5	6.3	14	18.2
3‐4 years	7	8.8	2	2.6
4‐5 years	2	2.5	2	2.6
>5 years	7	8.8	12	15.6
Total	80	100	77	100

**Table 3:** Patient Characteristics

Variable	N	%
Age group		
0‐12 months	47	58.8
13‐24 months	7	8.8
Over 24 months	26	32.5
Referred to audiology by		
Pediatrician	44	55.0
Outside Otolaryngologist	19	23.8
Outside audiologist	7	8.8
Newborn Hearing Screen	2	2.5
Early Intervention Group	3	3.8
Rutgers Otolaryngologist	4	5.0
Neurology	1	1.3
Premature		
No	70	87.5
Yes	8	10.0
Not recorded	2	2.5
NICU Stay >7 Days		
No	66	82.5
Yes	12	15.0
Not recorded	2	2.5
Newborn Hearing Screen		
Not recorded	10	12.5
Pass	9	11.3
Refer	58	72.5
Not tested	3	3.8
Degree of hearing loss		
Normal	2	2.5
Mild	5	6.3
Moderate	9	11.3
Moderate‐severe	11	13.8
Severe	11	13.8
Profound	28	35.0
Unspecified	14	17.5
Click air		
No	9	11.3
Yes	71	88.8
Click bone		
No	45	56.3
Yes	35	43.8
Click polarity		
Alternating	1	1.3
Rarefaction	14	17.5
Condensing	15	18.8
Condensing + Rarefaction	11	13.8
Condensing + Alternating	1	1.3
All 3	2	2.5
None	36	45.0
Tone burst air		
0	16	20.0
1	14	17.5
2	9	11.3
3	13	16.3
4	28	35.0
Tone burst bone		
0	60	75.0
1	3	3.8
2	5	6.3
3	2	2.5
4	10	12.5

Although there are some differences among the protocols, all recommend at least 2 tone burst frequencies be assessed by both air and bone conduction. This was completed in 21.2% of outside ABRs when applying the AAA/ASHA testing protocol. 3.7% of patients had complete ABR when applying the JCIH testing protocol (Table 4).

**Table 4:** Proportion and Corresponding Percentage of Patients with Missing Components of ABR Broken Down by Test Parameter and Guideline

ABR guidelines/protocol	Tone burst bone <2 F	Tone burst air <2 F	Click air absent	Polarity (at least 1)	Complete ABR per guidelines
JCIH Proportion of Patients (%)	63/80 (78.7%)	30/80 (37.5%)	9/80 (11.3%)	36/80 (45.0%)	3/80 (3.7%)
AAA/ASHA Proportion of Patients (%)	63/80 (78.7%)	30/80 (37.5%)	N/A	N/A	17/80 (21.2%)

Abbreviations: F, frequencies; N/A, not applicable to specified guideline/protocol.

An analysis of the individual testing parameters of all 80 ABR reports was then performed to determine which parameters most commonly led to an incomplete ABR by their respective protocol (Table 4). 78.7% of studies had fewer than 2 frequencies of tone burst bone conduction tested as specified by the JCIH and AAA/ASHA guidelines. 37.5% of studies had less than 2 frequencies of tone burst air conduction tested as specified by the JCIH and AAA/ASHA protocols.

Thirty‐seven patients experienced a change in diagnosis after completing their repeat ABR (Table 5). Unspecified diagnosis changed to sensorineural hearing loss (SNHL) after repeat ABR (N = 8) was the most common change, followed by a change in the degree of hearing loss (N = 7) and a mixed hearing loss to SNHL change (N = 4). 16.2% of the patients with a diagnosis change were newly diagnosed with normal hearing after the repeat ABR (average degree of originally diagnosed hearing loss: moderate), while 5.4% of the patients with changes were originally classified as normal hearing prior to their repeat ABR. Twenty‐three of the 37 patients with a change in diagnosis (62.2%) were recorded as having a change in treatment after their repeat ABR (Table 6). Most changes in treatments were from monitoring the patient's hearing to BAHA (N = 7) or cochlear implant (N = 6). Three patients had a change in treatment to either monitoring or no treatment after repeat ABR, and 17 patients changed from monitoring or no treatment.

**Table 5:** Patients Who Had a Change in Diagnosis Following Second ABR

Diagnosis change	Number (N = 37)	% of Changed diagnoses	% of Total patients
Unspecified to SNHL	8	21.6	10.0
Change in Degree	7	18.9	8.8
MHL to SNHL	4	10.8	5.0
SNHL to Normal	3	8.1	3.8
CHL to SNHL	2	5.4	2.5
MHL to CHL	2	5.4	2.5
Normal to CHL	2	5.4	2.5
SNHL to ANSD	2	5.4	2.5
Unspecified to Normal	2	5.4	2.5
CHL to MHL	1	2.7	1.3
MHL to Normal	1	2.7	1.3
Microtia to CHL	1	2.7	1.3
Unspecified to CHL	1	2.7	1.3
Unspecified to MHL	1	2.7	1.3

Abbreviations: ANSD, Auditory Neuropathy Spectrum Disorder; CHL, conductive hearing loss; change in degree, change in degree of hearing loss; MHL, mixed hearing loss; SNHL, sensorineural hearing loss.

**Table 6:** Patients Who Had a Change in Treatment Due to Repeat ABR

Change in treatment	Number (N = 23)	% of Changed diagnoses
CI to BAHA	1	2.7
Monitor to BAHA	7	18.9
Monitor to CI	6	16.2
Monitor to HA	1	2.7
Monitor to none	1	2.7
Monitor to pending	1	2.7
HA to CI	1	2.7
HA to Monitor	1	2.7
HA to BMT	1	2.7
HA to None	1	2.7
None to BAHA	1	2.7
Speech therapy to CI	1	2.7

Abbreviations: BAHA, bone anchored hearing aid; BMT, bilateral myringotomy with tubes; CI, cochlear implant; HA, hearing aid; None, no intervention required.

## Discussion

Accurate and complete ABR testing is essential to properly diagnose and manage hearing loss in pediatric patients, including infants.^12^, ^13^ This study demonstrated that despite the accepted and published 2007 JCIH, 2011 AAA, and 2012 ASHA testing guidelines, very few children who presented to our facility between 2012 and 2019 had complete ABR testing as defined in this study. Incomplete ABR studies prohibit Otolaryngologists from being able to clear the child for amplification and recommend appropriate additional testing (ie, imaging) during the initial Otolaryngology visit. When a study is incomplete, testing must be repeated either utilizing behavioral audiometry or via ABR (possibly under sedation), and a second office visit with Otolaryngologists is necessary before the child can be medically cleared for amplification. The recent Early Hearing Detection and Intervention (EHDI) change from a 1‐, 3‐, and 6‐month approach to screening‐diagnosis‐intervention to a 1‐, 2‐, and 3‐month model further highlights the need for diagnostic ABR studies to be completed before evaluation by Otolaryngologists.^3^

This study contained a high proportion of patients with severe to profound hearing loss. The patients who are referred to a tertiary care center for hearing evaluation are most likely those with significant hearing loss requiring possible cochlear implantation or other surgical therapies. The relatively high number of patients diagnosed with unspecified hearing loss calls into question what factors were missing in their first ABR testing to limit a definitive diagnosis being made. The remainder of this paper discusses what factors were missing in the first ABR testing to result in a potentially inaccurate hearing loss diagnosis.

The number of tone burst frequencies assessed is critical. Over interpolation of threshold levels at specific frequencies may lead to over or under amplification when hearing aids are programmed using DSL and NAL‐NL1 targets which provide different gain by frequency.^15^ Numerous studies have shown the agreement in tone burst‐evoked ABR thresholds being used to estimate pure‐tone thresholds in order to make an accurate hearing loss diagnosis.^4^, ^16^, ^17^, ^18^, ^19^, ^20^, ^21^, ^22^ Tone burst testing is therefore an essential testing component of the standard ABR as the JCIH, AAA, and ASHA all require some level of tone burst threshold testing.^7^, ^8^, ^9^

Thirty‐one percent of the ABR studies used a bone conduction click to estimate bone conduction thresholds. Some studies have found that click‐evoked ABRs can be used to predict pure‐tone behavioral thresholds.^23^ However, other studies have found that click stimuli can result in threshold levels appearing lower than they are.^17^, ^22^, ^24^ This is because click stimuli are broadband, thereby stimulating a larger portion of the basilar membrane.^17^ The 2020 AAA testing protocol clarifies that a bone conduction click should not be used to substitute a tone burst bone conduction testing for the same rationale of inaccurate threshold testing.^25^

Inadequate use of polarity was another common factor contributing to an incomplete ABR. The main difference between the JCIH and AAA/ASHA protocol is polarity testing. This most likely contributed to the further decrease in the number of complete ABRs for JCIH protocol (3.7%) when compared to AAA/ASHA protocol (21.2%). Polarity testing is the only way to assess for Auditory Neuropathy Spectrum Disorder (ANSD). It is conducted by performing either a bin reviewable alternating click or both condensing and rarefaction click responses. Failure to perform a two‐polarity click may lead to incorrect identification of waves I, III, and V.^10^, ^11^

Lastly, requiring raw waveform data to be available can be critical for interpreting the ABR for visual confirmation of the reported results and agreement with the Audiologist performing the exam. Both the 2020 AAA and 2021 ASHA recommend, but do not require, including waveforms in the final report. The ability for a second Audiologist or Otolaryngologist to review raw waveforms may reduce the need to repeat the study if updated behavioral audiometry is not in agreement with ABR findings or if ANSD is suspected, ultimately saving time and unnecessary sedation for future ABRs.

Inadequate or incomplete ABR testing can lead to incorrect initial diagnoses, ultimately impacting early treatment of patients. In this study, nearly half of the patients analyzed had a change in diagnosis after their repeat ABR was conducted. Changes in management of these patients from monitoring to necessitating either BAHA or cochlear implant were the most common changes noted, which further highlights concern for delayed treatment after no active management was prescribed following the initial ABR. Previous literature has shown that delays in receiving these treatments are associated with slowed expressive language and vocabulary development in children.^26^, ^27^ Tomblin et al found that expressive language growth was faster in patients who received cochlear implants as infants versus those who received implants as toddlers, and they attributed 14.6% of the variance individual differences in these growth rates to age at initial stimulation.^27^ Dettman et al. determined when comparing speech perception, language acquisition, and speech production accuracy among children under 6 years old who received cochlear implants for congenital bilateral hearing loss, those that received them when under 12 months old had significantly higher scores than older children.^26^ By not having a correct diagnosis for children in part due to incomplete or inadequate ABRs, they are increasingly susceptible to negative outcomes in their language development. Furthermore, even in cases where treatment strategies changed to no treatment or monitoring after repeat ABR, these cases still cause unnecessary initial treatment and wasted healthcare costs and allocation of resources, such as appointment slots and physician's time. Therefore, incomplete ABRs that inaccurately diagnose newborns not only can lead to worse patient outcomes but also can cause increased healthcare costs and resources wasted.

The authors acknowledge in clinical practice there are other considerations that may lead to an incomplete ABR testing, including time. Sedated ABR study may be limited due to the child's exposure to anesthesia. Unsedated ABR study is also limited by the child waking up during testing. These constraints may lead to missing data and incomplete testing. It is the opinion of these authors that such constraints be clearly specified in the ABR report, and every effort should be made to continue testing in a second session, if necessary. None of the outside ABR reports recommended testing to be repeated at a second session to achieve a complete study. It is equally important for both the Otolaryngologist and Pediatrician to determine which patients can safely undergo a sedated ABR procedure with benefits outweighing the risks.

**Figure 1:**
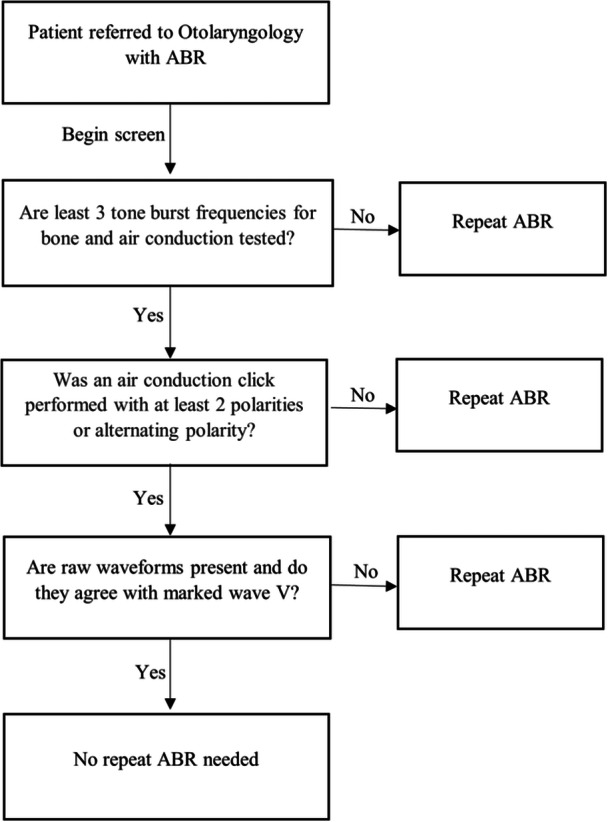
Screening guidelines for a complete ABR for Otolaryngologists.

Otolaryngologists routinely rely on ABR testing to determine degree and type of hearing loss to recommend appropriate management in their patients. As shown, there are many incomplete ABR tests being done, and these patients are then referred for evaluation by an Otolaryngologist. In addition, most of the children in this study who were referred to Rutgers/UH Department of Audiology were referred by their Pediatricians after the first ABR. Most patients were referred to the UH Department of Audiology as opposed to Department of Otolaryngology, which aligns with the recommended approach algorithm in pediatric clinical guidelines.^28^ However, if Pediatricians are directly referring patients to an Otolaryngologist, it may be necessary for stringent screening of these studies by the Otolaryngologist to determine if the study is complete or additional information is needed to determine appropriate management. To help screen efficiently and quickly for incomplete ABR testing, we propose a brief screening guide for Otolaryngologists to determine the need for a repeat ABR (Figure 1). This workflow proposal is based on the most common reasons for an incomplete ABR identified from this study.

Limitations of this study include the relatively small sample size of 80 ABR tests, and possible bias from geographic barriers as the studies were conducted in New Jersey. This was a retrospective chart review and as such a common limitation is missing data. Fortunately, there was no missing data for the included patients in this study. Additionally, newer guidelines such as the 2019 JCIH and 2020 AAA testing protocols are now available, but since the years of this study covered 2012 to 2019, only guidelines that were applicable at the time were used.^3^, ^25^ However, these newer guidelines have not changed substantially and the screening guide in Figure 1 remains applicable. Future studies could also be performed to determine if ABR studies are found to be incomplete across the United States, as opposed to solely New Jersey. This further emphasizes the need for establishing more specific ABR testing protocol for Audiologists performing the testing, and ABR result interpretation/screening protocol for Otolaryngologists reviewing them to recognize what constitutes a complete ABR and when a repeat ABR should be ordered.

## Conclusion

ABR testing is the gold standard for diagnosing hearing loss in pediatric patients under 3 years of age, and the only measure for children 6 months of age and younger. This study demonstrates that many infants and children have incomplete ABR tests performed that could potentially lead to misdiagnosis, delays in diagnosis, and inappropriate interventions. The most common changes in interventions from initial to repeat ABR were those that now required cochlear implants or BAHA. We propose an ABR screening workflow for Otolaryngologists to recognize what constitutes a complete ABR testing to help identify the need to refer for a repeat ABR to obtain an accurate diagnosis and avoid delay in patient care.

## Author Contributions

**Hannaan S. Choudhry**, design, data analysis and interpretation, revisions, writing; **Roman Povolotskiy**, design, data analysis and interpretation, writing; **Shahin Damji**, design, data analysis and interpretation, writing; **Yu‐Lan M. Ying**, design, data analysis and interpretation, writing, review and editing, project supervision. **Nicole Raia**, design, writing, review and editing, project supervision.

## Disclosures

### Competing interests

None.

### Funding source

None.
